# The educational value of ward rounds for junior trainees

**DOI:** 10.3402/meo.v20.27559

**Published:** 2015-04-21

**Authors:** Faidon-Marios Laskaratos, Deirdre Wallace, Despoina Gkotsi, Aine Burns, Owen Epstein

**Affiliations:** 1 Centre for Gastroenterology, Royal Free London NHS Foundation Trust, London, United Kingdom; 2 Academic Centre for Medical Education, University College London, London, United Kingdom; 3 Royal College of Physicians, London, United Kingdom; 4 Department of Ophthalmology, Moorfields Eye Hospital, University College London, London, United Kingdom; 5 Sheila Sherlock Education Centre, Royal Free London NHS Foundation Trust, London, United Kingdom

**Keywords:** ward round, postgraduate education, foundation training

## Abstract

The ward round (WR) is a complex task and medical teachers are often faced with the challenge of finding a balance between service provision and clinical development of learners. The educational value of WRs is an under-researched area. This short communication aims to evaluate the educational role of WRs for junior trainees and provides insight into current practices. It also identifies obstacles to effective teaching/training in this setting and provides suggestions for improving the quality of WR teaching.

The ward round (WR) is a complex task which has been described as ‘walking a tightrope’. The medical teacher needs to balance service demands with the educational needs of learners and often has to teach multiple learners who have different learning objectives (1). The rotational nature of training and the shift-work pattern of learners add to the complexity of this educational activity (2).

The educational value of WRs for doctors in training is an under-researched area. There are a limited number of original research papers in the literature. Most of these studies have relied on questionnaire (3–6) or audit (2) data. They have been performed in considerably different settings (e.g., countries, hospitals, assessing different types of WRs) and have involved different participants (learners at different levels of training, teachers), which may largely account for the contrasting results about the educational value of WRs in the development of trainees’ skills (7).

## Methods

Our study was designed to investigate the educational value of WRs for Foundation Year 1 & 2 trainees. The setting was a large teaching hospital in London, United Kingdom.

A voluntary and previously piloted paper questionnaire (Supplementary file) was distributed to foundation trainees during a scheduled mandatory weekly teaching session in the middle of the academic year, so that trainees had been exposed to WRs for a reasonable period. Absent trainees were contacted via e-mail by the Education Centre and up to one reminder was sent to non-responders to improve response rates. The questionnaires were collected anonymously.

The questionnaire was divided into several sections (demographic data, characteristics of WRs, learning opportunities, obstacles to effective learning, areas for improvement, and characteristics of successful WRs), which covered many different aspects of teaching and learning on WRs. Qualitative questionnaire data were organised in codes and themes by two authors (FL, DG) independently and any discrepancies were resolved by discussion and mutual agreement.

The study protocol was reviewed by the Academic Institution's Ethics Screening Service and did not require ethical approval through the Academic Institution's Research Ethics Committee.

## Results

A total of 40 of 95 foundation trainees at our hospital returned the questionnaire (42% response rate). Of those, 25 were Foundation Year 1 doctors and 15 were Foundation Year 2 trainees. There was a balanced mixture of Foundation Year trainees with regards to sex (male=19, female=21) and specialty representation (medical specialties=17, surgical specialties=17, other=6).

Most trainees participated in five registrar- or consultant-led WRs per week (range: 2–7). The average duration of a WR was 134 min (range: 15–300) and on average 9 min was reportedly spent with each patient (range: 2–20). Of the responders, 42.5% felt that WRs were totally service orientated without any teaching, 35% mentioned that there was some teaching during the WR, and 22.5% reported that there was a mixture of WRs, some with and some without teaching. Little time was devoted to teaching during the WR and a significant amount of time was spent on administrative tasks. Trainees also mentioned that seniors rarely asked questions on WRs or gave feedback. Juniors described only limited opportunities to ask questions, present patients, or learn new material.

The educational value of WRs in the development of different skills based on questionnaire responses is summarised in Table 1. WRs were generally considered useful in knowledge acquisition, selection, and interpretation of diagnostic investigations, patient management, record keeping, and approach towards patients. In contrast, they were not considered as useful in developing history taking, physical examination, leadership skills, or in learning ethical principles.

**Table 1:** Questionnaire data (trainee responses): learning opportunities on WRs

Skills learned	1 (=not beneficial)	2	3	4	5 (=extremely beneficial)
Knowledge	4	9	11	13	3
History	7	18	9	5	1
Examination	6	12	12	9	1
Investigations	1	8	15	11	5
Patient management	0	5	12	14	9
Communication	8	7	14	9	2
Time management	4	10	15	9	2
Record keeping	3	6	7	19	5
Team working	3	6	11	16	4
Presentation	8	8	10	12	2
Leadership	9	14	10	6	1
Ethics	6	18	7	7	2
Approach to patient	4	8	9	14	5

Note: WRs were perceived to be beneficial mainly in the development of skills that are highlighted in yellow.

Absolute numbers out of 40 respondents.

Trainee responses ranking learning events in terms of their contribution to their total learning are presented in Table 2. Textbooks, online resources, and lectures were ranked higher in terms of educational value, compared to WRs, whereas journals and conferences were ranked lower.

**Table 2:** Questionnaire data (trainee responses): ranking of learning events in terms of their contribution to trainee's total learning and obstacles to effective learning

	Rank					
**Learning event**	**1 (=most useful)**	**2**	**3**	**4**	**5**	**6 (=less useful)**
Textbooks	10	9	6	10	2	3
WR	6	7	6	6	7	8
Journals	4	6	5	4	14	7
Lectures	9	7	11	10	2	1
Online resources	10	7	5	7	7	4
Conferences	1	4	7	3	8	17
**Obstacle**	**1 (=strongly disagree)**		**2**	**3**	**4**	**5 (=strongly agree)**
Lack of time	1		0	2	11	26
Number of patients	0		2	6	12	20
Interruptions	0		0	3	12	20
No interest from seniors	0		6	14	10	10
Bedside crowding	4		8	15	6	7
Patient factors	8		9	17	5	1
Ward environment	2		10	15	12	1
Team structure	4		10	15	6	5
Over-reliance on technology	12		16	6	4	2

Note: Factors perceived as significant obstacles to effective learning on WRs are highlighted in yellow, whereas factors perceived as minor obstacles are highlighted in red.

Absolute numbers out of 40 respondents.

Trainees considered lack of time, large number of patients, frequent interruptions, and lack of interest from seniors as the main obstacles to effective teaching and learning on WRs. In contrast, patient factors, such as compliance or availability, and over-reliance on technology were not perceived as important obstacles (Table 2).

Invited suggestions for improving the quality of teaching on WRs are summarised in Table 3. These relate mainly to changes in the role of juniors and seniors on the WR, the structure of rounds, and the availability of adequate time dedicated to teaching either during the round or in pre-or post-WR meetings.

**Table 3:** Questionnaire data (trainee responses): suggestions for improvement of teaching on WRs

Common themes (and codes) were:
Role of seniors - More senior interest - Seniors to ask questions - Seniors to give formal feedback on performance
More time devoted to teaching - Spending longer on each patient - Consultant to explain rationale for decisions - Departmental teaching based on cases seen, such as ‘morning report’ - Consultants to teach whilst reviewing X-rays
Separate teaching rounds - One WR per week that is more dedicated to teaching
Presence of two seniors on WR: - One to teach, the other to concentrate on the WR - Divide the WR in a teaching WR and a service WR
Role of juniors - Juniors to present patients - Juniors to examine patients with signs - Juniors to look things up and present to team - Juniors to lead the WR under close supervision - Juniors to suggest management plan - More opportunity to examine and clerk patients rather than just scribe during the WR or be sent to do jobs
Debrief after WR (with some take-home messages)

The majority of trainees reported that learning atmosphere, clinical teaching, teaching style, communicating expectations, and team management are all important characteristics of successful WRs (data not shown).

## Discussion

This study demonstrated that almost half the trainees who responded reported WRs to be service orientated with little time devoted to teaching. WRs were perceived to be useful in the development of certain skills, such as patient management and investigations, but less useful in other skills, such as physical examination. The main obstacles to effective learning and teaching were lack of time, number of patients, frequent interruptions, lack of interest from seniors, and the relationship between seniors and juniors. Many suggestions were made to improve the quality of teaching on WRs, including changes in the role of juniors and seniors, the structure of the WRs, and the introduction of formalised teaching sessions (e.g., pre- or post-WR sessions). Finally, learning atmosphere, clinical teaching, teaching style, communicating expectations, and team management were all considered important characteristics of successful WRs.

Our study findings are similar to those of other studies. Claridge (8) reported that only 9% of an average WR is devoted to teaching and only 36% of foundation trainees felt that WRs were a good learning opportunity. Chaponda et al. (2) in their study of the educational value of post-take WRs concluded that, although NHS targets are met, junior doctors’ education is compromised with the introduction of the European Working Time Directive (EWTD) and Modernising Medical Careers (MMC).

Claridge (8) reported that only 27% of foundation doctors agreed that WRs were a good opportunity to learn physical examination and only 36% agreed that they were a good learning opportunity to learn history taking. In contrast, 76% mentioned that they were a good opportunity to learn diagnostic investigations. These findings are similar to those of our study and may reflect a shift away from using history and examination towards using investigations to reach a diagnosis. Tariq et al. (3) reported that trainees felt patient management was the aspect best covered on WRs, whereas teaching of clinical skills and bedside examination was the weakest aspect (9).

Likewise, in terms of obstacles to effective teaching and learning on WRs, our findings are similar to those published by Claridge (8) who reported that lack of time, number of patients, and team structure were the main factors. Claridge (8) also maintained that junior doctors need to become part of ‘a community of practice’ to maximise their learning and that frequent rotations allow less time to build advantageous clinical relationships.

Many of the suggestions for maximising learning on WRs identified in our study are in keeping with the findings of other studies. Stanley (9) mentioned that learning opportunities could be created by structuring WRs and including formal discussion times into the WR or in pre- and post-WR meetings. Ali Abdool and Bradley (10) agreed that structuring WRs (e.g., preparation phase, using a roadmap, debriefing) is important, as is the role of seniors, who should communicate their expectations, provide a focus for each encounter, and give prompt feedback.

It is clear that if students and doctors in training are not prepared for their teaching role, the quality of WR teaching will be diminished. As a result, many accreditation bodies focus specifically on whether medical teachers are prepared to teach and give feedback. For example, the Liaison Committee on Medical Education (LCME), which is responsible for the programmatic accreditation of medical education programmes leading to the MD degree in the United States and Canada, emphasises the importance of medical teachers (e.g., residents, postdoctoral fellows, faculty members) being prepared for their roles in teaching and assessment. Similarly, in the United Kingdom, the General Medical Council (GMC), which is responsible for the accreditation of medical schools and for the quality assurance of the Foundation Programme training, focuses on the management of teaching, learning, and assessment, as outlined in relevant documents (*Tomorrow's Doctors* and *Quality Assurance of the Foundation Programme*) (11–13).

In terms of characteristics of successful WRs, Roy et al. (1) conducted a multi-centre study enrolling students, residents, and faculty and identified that learning atmosphere, clinical teaching, teaching style, communicating expectations, and team management are essential features of successful WRs.

Our study has several limitations. First, it is a single centre study in a large and busy teaching hospital in London which may limit its applicability to other settings. However, other similar studies, which have been performed both in the United Kingdom and internationally, have produced findings which are in keeping with many of those reported in our study. This provides further validity to our conclusions and suggests that the decline in the quality of teaching and learning is a general characteristic of busy WRs. The corresponding author is currently conducting another medical education study in association with University College London and the Royal College of Physicians of London, which is investigating the educational value of post-take WRs for medical higher specialty trainees (senior residents) in a busy district general hospital in London, using questionnaire surveys and semi-structured interviews for triangulation purposes. Interestingly, our provisional data demonstrate that, despite some differences due to the higher level of trainees and their different educational objectives, many obstacles and issues relating to teaching and learning on busy WRs are similar, highlighting the generalisability of our results in other settings, where the high volume of workload that trainees and medical teachers are faced with, may impact on the quality of teaching and learning. Another limitation is that our study identified perceptions of learners. It was not an observational study and therefore may not be an accurate reflection of practices on current WRs. It also did not triangulate the findings by identifying perceptions of teachers. However, our study has many strengths. Our questionnaire covered many areas of teaching and learning on WRs and is one of the most comprehensive questionnaires in the available literature looking into this important topic. It is also one of the few studies investigating the educational value of WRs for junior trainees after the introduction of recent changes in postgraduate medical education in the United Kingdom, such as the Foundation Programme in 2007 and the implementation of the EWTD and MMC.

## Conclusions

The educational value of WRs for junior doctors is an under-researched and underused area of postgraduate medical education. The present study has contributed to knowledge in this area by increasing the understanding of current practices about learning opportunities on WRs, as well as exploring the perceived obstacles to effective WR teaching and learning. It also offered a variety of realistic suggestions for improving the quality of teaching on WRs and maximising the benefit of what is an underused but potentially very powerful education tool.

## Supplementary Material

The educational value of ward rounds for junior trainees
