# Supplementary material for: The educational value of ward rounds for junior trainees
Source: Med Educ Online. 2015 Apr 21;20:10.3402/meo.v20.27559. doi: 10.3402/meo.v20.27559 (PMC4408315; doi:10.3402/meo.v20.27559)
Supplement: The educational value of ward rounds for junior trainees [file MEO-20-27559-s001.pdf]

Appendix 1

Part 1: Demographic data

Please tick the correct box:

1. Grade: F1□ F2□

2. Sex: Male □ Female□

3. Current rotation:

Medicine□ Surgery□ Other□ …………(please specify)

(“Other” category: if your rotation does not have ward rounds, then please consider your last rotation where ward rounds were available and mention if this was a medical or surgical rotation in brackets)

Part 2: Characteristics of ward rounds (WRs)

Please answer the following questions:

1. On how many consultant/SpR-led WR per week do you take part? __

2. What is the average duration of a WR (in mins)? __

3. How much time on average do you spend with each patient on the WR (in mins)? __

	None	Small amount	Moderate amount	Significant amount
4. How much time during an average WR is devoted to <u>teaching</u> (both formal and informal)?				
5. WRs contribute in how much of your <u>total learning</u>?				
6. How much time during the WR is spent on <u>routine administrative tasks</u> (such as, organising investigations, finding forms, completing paperwork etc)?				

7. Do you regard the WR in your hospital as: (please circle)

a. A ward round with no teaching at all, focusing on service only

b. A ward round which incorporates some teaching into service provision

c. a mixture of both

8. Please tick the relevant box:

	Never	On few WRs	On some WRs	On most WRs	On every WR
I have opportunities to ask questions					
The consultant/SpR asks me questions					
I present patients on the WR					
I learn something new on the WR					

I receive feedback on my performance					

Part 3: Learning opportunities

Please rate (1=not at all beneficial, 5=extremely beneficial) how beneficial WRs are in teaching you the following skills by ticking the relevant box:

Skills learnt	1 (=not beneficial)	2	3	4	5 (=extremely beneficial)
Conveying medical knowledge					
History taking					
Physical examination					
Diagnostic investigations					
Patient management					
Communication skills					
Time management					
Record keeping					
Teamworking					
Presentation skills					
Leadership skills					
Ethical principles					
Approach towards patients					

Please rank the following learning events in terms of their educational value (1=most useful, 6= less useful):

Learning event	Rank
Reading textbooks	

Ward round	
Reading journals	
Lectures/presentations (such as Grand Round, weekly Foundation Programme mandatory teaching etc)	
Online educational resources (such as BMJ learning, Pastest, Onexamination etc)	
Conferences	

Part 4: Obstacles to effective learning

Which of the following factors are making learning on WRs problematic? (please tick the relevant box)

Factor	1 (=strongly disagree)	2	3	4	5 (=strongly agree)
Lack of time					
Large number of patients					
Interruptions (eg bleeps/ having to leave the WR to organise investigations or find forms etc)					
Lack of interest from seniors					
Bedside crowding					
Patient factors (eg compliance, meal times, not being available)					
Ward environment (eg busy, noisy)					
Team structure					
Over-reliance on technology					
Other……………………….					

Part 5: Areas for improvement

Please make 3 suggestions to improve the quality of the teaching on WRs:

1)

2)

3)

Part 6: Characteristics of successful ward rounds

Please rate the following factors in terms of their importance in making WRs an educationally valuable experience for you: (please tick the relevant box)

Factor	Examples	1 (=strongly disagree)	2	3	4	5 (=strongly agree)
Learning atmosphere	The consultant/ SpR is:<br>-approachable<br>-Ethusiastic towards teaching<br>-Gives you feedback<br>-not in a rush					
Clinical Teaching	The consultant/ SpR:<br>-Is a role model<br>-Shares his/her thought processes<br>-Sets aside time to teach<br>-Discusses relevant evidence-based studies/literature					

	-Looks holistically at the patient					
Teaching style	The consultant/ SpR:<br>-Asks questions<br>-Demonstrates key physical findings<br>-Creates succinct teaching points<br>-Allows trainee involvement (eg to ask questions/present cases)					
Communicating expectations	The consultant/ SpR:<br>-States what is expected of trainees during the WR<br>-Allows team some independence in decision making<br>-Sets feasible teaching and learning goals					
Team management	The consultant/ SpR:<br>-Conducts WRs in an organised, efficient and timely fashion<br>-Can maintain interest in all members of the team<br>-Has a consistent, coherent plan of care					
